# Aortic Stiffness and Alzheimer’s Disease: The Medin Connection

**DOI:** 10.3390/biom15081148

**Published:** 2025-08-08

**Authors:** Filippos Triposkiadis, Andrew Xanthopoulos, Harisios Boudoulas, Dirk L. Brutsaert

**Affiliations:** 1 European University Cyprus, 1516 Nicosia, Cyprus; 2 Department of Cardiology, University Hospital of Larissa, Faculty of Medicine, School of Health Sciences, University of Thessaly, Biopolis, 41500 Larissa, Greece; 3 Division of Cardiovascular Medicine, The Ohio State University, Columbus, OH 43210, USA; 4 Department of Medicine & Cardiology, University of Antwerp, 2000 Antwerpen, Belgium

**Keywords:** aging, aorta, stiffening, Alzheimer’s, medin, hypothesis

## Abstract

Aging is associated with aortic stiffening (AoSt), a condition characterized by diminished aortic elasticity that predisposes individuals to cognitive decline, including Alzheimer’s disease (AD). Emerging evidence implicates medin, which is derived from milk fat globule-EGF factor 8 protein (MFG-E8), as a key link between AoSt and AD. Medin aggregates into aortic medial amyloid (AMA), which is found in approximately 97% of Caucasian individuals aged 50 and above, contributing to vascular inflammation, calcification, and loss of arterial elasticity. These changes may promote hyperpulsatile cerebral blood flow and impair glymphatic clearance, resulting in increased deposition of neurotoxic proteins, such as amyloid-β (Aβ) and possibly medin, which colocalizes with vascular Aβ in the brain. Medin enhances Aβ aggregation, generating heterologous fibrils, and thereby contributes to cerebrovascular dysfunction and neuroinflammation. This interaction (cross-seeding) may deteriorate amyloid pathology in both the vasculature and the parenchyma in AD. Furthermore, medin per se causes endothelial dysfunction, increases oxidative stress, and activates glial cells, promoting the development of a pro-inflammatory environment that enhances cognitive decline. In this manuscript, we contend that medin might act as a bridge connecting the age-related increase in aortic stiffness to AD, and therefore, medin might present a novel therapeutic target within this context. This hypothesis deserves experimental and clinical validation.

## 1. Introduction

Aging is characterized by aortic stiffening (AoSt), the limited capacity of the aorta to expand and recoil as result of oscillations in pressure [1]. AoSt has been linked to several morbidities that predispose individuals to focal or global cerebral dysfunction, such as systolic hypertension [2,3]. Increased left ventricular (LV) afterload associated with reduced coronary perfusion pressure promotes LV remodeling [4,5] and kidney damage [6,7]. Importantly, several of these morbidities often coexist in the same individual [8].

The accumulation of amyloid fibrils seems to be universal in the elderly [9]. Aortic medial amyloid (AMA) is the most common human amyloid, as it has been found in approximately 97% of Caucasian individuals above 50 years of age [10]. AMA is derived from a proteolytic fragment of milk fat globule-EGF factor 8 protein (MFG-E8), also known as lactadherin [11]. Medin, a peptide consisting of 50 amino acids that is cleaved from the second discoidin-like domain of MFG-E8, constitutes the primary component of AMA and shares 16% of its sequence with amyloid β (Aβ) [12,13], which accumulates in Alzheimer’s disease (AD).

AD is a debilitating neurodegenerative disease that affects millions of individuals globally, and is characterized by memory loss, cognitive decline, and behavioral disturbances [14]. Two types of brain lesions define the neuropathological diagnosis of AD [15]. The first type, *Aβ plaques*, has become a unifying term for heterogeneous formations, many of which contain massed, fibrillar polymers of Aβ, but some of which lack the defining amyloid features [16]. The second type, intracellular *neurofibrillary tangles*, contains tau protein as the primary protein constituent, which is critically essential for the manifestation of dementia [17].

Accumulating evidence supports the association of AoSt with dementia-related pathology [18,19,20,21,22,23] and contributes to the pathogenesis of AD [24]. In this manuscript, we summarize the structure and function of the young and aging aorta, and contend that medin may be the link between increased AoSt and the development of AD in the elderly.

## 2. The Effect of Aging on the Aorta

### 2.1. The Young Aorta

The wall of the aorta comprises three main layers: the tunica intima, tunica media, and tunica adventitia [25]. The internal elastic lamina separates the tunica intima from the tunica media, whereas the external elastic lamina separates the tunica media from the tunica adventitia.

The endothelium of the intima, which lies between the circulating blood and the underlying cellular milieu, is a key mediator of cardiovascular function and displays transcriptional plasticity in response to normal and abnormal stimuli [26,27]. The endothelium not only serves as a barrier, but additionally actively regulates organ development, homeostasis, and tissue regeneration [28,29,30]. Studies employing single-cell RNA sequencing have revealed the presence of specialized subpopulations of endothelial cells (ECs) with distinct marker genes, suggesting functional specialization [31]. ECs sense various stimuli, including fluid shear stress and cytokines, and actively control the contraction and relaxation of vascular smooth muscle cells (VSMCs) located in the media through the secretion of various mediators [32].

**Figure 1:**
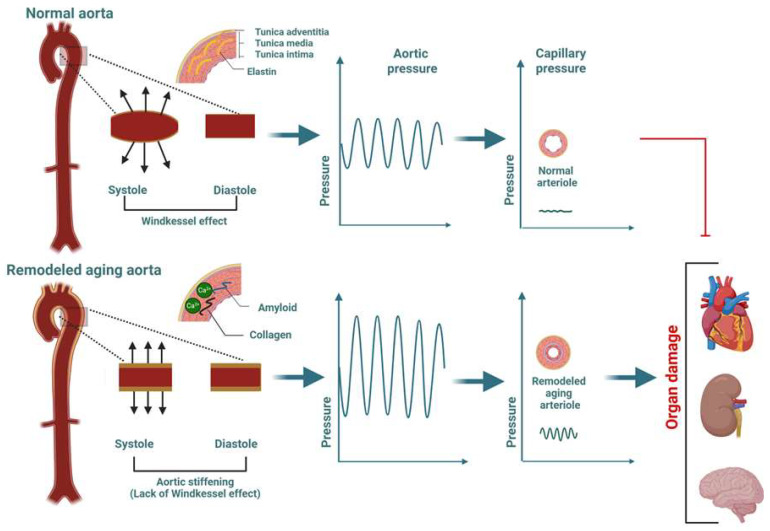
In the young, a biomechanically normal functioning ascending aorta expands during left ventricular (LV) systole and recoils during LV diastole, allowing vessel energy storage from LV systole and energy redistribution during LV diastole through elastic recoil, thereby providing antegrade flow. This phenomenon, *the Windkessel effect*, is crucial for the maintenance of pulse pressure within the physiological range. The hallmark of the aging aorta is remodeling of the extracellular matrix (ECM), including the dissolution of elastic fibers and accumulation and cross-linking of collagens. Other characteristic features include vascular calcification and medin amyloid deposition, predominantly in the tunica media. These structural abnormalities develop in the setting of an inflammatory environment (inflammaging) and eventually lead to enhanced aortic stiffness (AoSt) in the elderly. Elevated AoSt augments the LV pulsatile load (elevation of systolic blood pressure (BP), decrease in diastolic BP, and widening of pulse pressure), which is transmitted in the microvasculature, contributing to organ damage, particularly to the heart, the kidney, and the brain. Created in BioRender (https://www.biorender.com/).

The aortic tunica media, which virtually determines aortic biomechanics [33], consists of VSMCs and an extracellular matrix (ECM) containing elastic lamellae and discontinuous basement membrane components, along with interstitial collagens [34] with a specialized structure. Elastin dominates the mechanical behavior of aortic tissue under normal pressure fluctuations, working together with VSMCs of the contractile phenotype to allow the large aortic deformations necessary for cardiovascular functioning. The collagen does not contribute to biomechanics until a high deformation level is reached, which is assumed to exceed the level of physiological functioning. A biomechanically normal functioning ascending aorta distends during LV systole and recoils during LV diastole, allowing the storage of energy in the vessel from LV systole and energy redistribution during LV diastole by elastic recoil, which provides antegrade flow [35]. This phenomenon, the *Windkessel effect*, is crucial for the maintenance of pulse pressure within the physiological range (Figure 1).

In healthy young adults, the LV–aortic interactions are optimized to deliver cardiac output with a suitable pulsatile hydraulic load on the LV [36,37]. The low-impedance, compliant aorta interconnects with conduit arteries, such as the carotid, which have higher stiffness. The resulting impedance mismatches, accompanied by wave reflections at the aorta–brain interfaces, restrict the transmission of immoderate pulsatile energy into the cerebral microcirculation, protecting the brain tissue [38,39]. Besides impedance mismatch, other mechanisms contributing to protection against excessive pulsatile energy transmission include vessel size and area [40,41]. Optimal pulsatile energy transmission to the brain occurs when the human heart rate is close to normal, and it is stable across a wide range of aortic arch stiffnesses and LV contractility [42].

### 2.2. The Aging Aorta

The hallmark of the aging aorta is ECM remodeling, including fragmentation of elastic fibers and excessive deposition of cross-linked collagens [43]. Vascular calcification and the presence of AMA, predominantly in the tunica media of the thoracic aorta and other upper-body arteries, are also characteristic features [44,45]. These structural abnormalities develop in the setting of an inflammatory environment (*inflammaging*) and eventually lead to enhanced AoSt in the elderly [41].

#### 2.2.1. Inflammaging

During aging, cells in the body undergo senescence, characterized by a dysfunctional phenotype, the so-called senescence-associated secretory phenotype (SASP) [46].

Senescent ECs are flattened and enlarged and exhibit increased polyploidy, decreased nitric oxide (NO) production, and increased secretion of several pro-inflammatory cytokines [47]. As a result, the senescence of ECs accelerates vascular aging by increasing intima media layer thickness, the deposition of collagen, and often the thickness of the vascular lumen, and by reducing elastin deposition [48].

Senescent VSMCs in the aging aorta secrete inflammatory cytokines, chemokines, growth factors, and factors of matrix remodeling that change the tissue environment and participate in the development of the chronic, sterile, low-grade inflammation that characterizes inflammaging [49,50]. The latter results in damage to or loss of elastic fibers through the recruitment of several processes (e.g., enzymatic degradation, oxidative damage, glycation, calcification, etc.), which act on top of mechanical fatigue [51]. Further, significant collagen changes occur in the aging aortic wall, including an increase in its amount and a change in its structure, as well as an increase in cross-linking between collagen fibers [52]. Inflammaging is accelerated by several factors, including sex, race, coexisting morbidities, such as hypertension, obesity, and diabetes mellitus, and lifestyle (e.g., excessive alcohol consumption, smoking, and physical inactivity) [53], and it is tightly linked with aortic calcification and AMA [54,55,56].

Several lines of evidence indicate that inflammaging promotes aortic media calcification by mediating the development of a VSMC synthetic phenotype [57,58]. Calcification occurs at the intima and the media of the arterial wall. Calcification of the intima is linked with atherosclerosis, whereas that of the media has been associated with arteriosclerosis, an age-associated disease [59]. Senescent VSMCs undergo calcification following the uptake of exosomes derived from the endothelium [60,61] or of microparticles [62] released upon endothelial stimulation by chemical messengers of inflammation. In this regard, TNF (tumor necrosis factor)-α stimulates endothelial cells to release microparticles that contain BMP2 (bone morphogenetic protein 2), which subsequently undergo phagocytosis by VSMCs and enhance osteogenesis [63]. CRP (C-reactive protein), another messenger of inflammation, has also been implicated in promoting age-associated VSMC osteogenic trans-differentiation [64]. Finally, DNA damage of diverse etiology facilitates the generation of reactive oxygen and nitrogen species [65,66], which, in turn, promotes the loss of VSMC contractile function and the enhancement of VSMC osteogenic differentiation and calcification [67].

#### 2.2.2. Medin

Experimental studies in mice have demonstrated that medin aggregates develop in the aorta and brain vasculature age-dependently, and that genetically induced deficiency in MFG-E8 abolishes vascular accumulation [68]. Medin has a high affinity to elastin and is tightly linked to arterial inflammation, dissolution of elastic fibers, calcification, and AMA generation [69].

##### Medin Amyloid Formation

**Figure 2:**
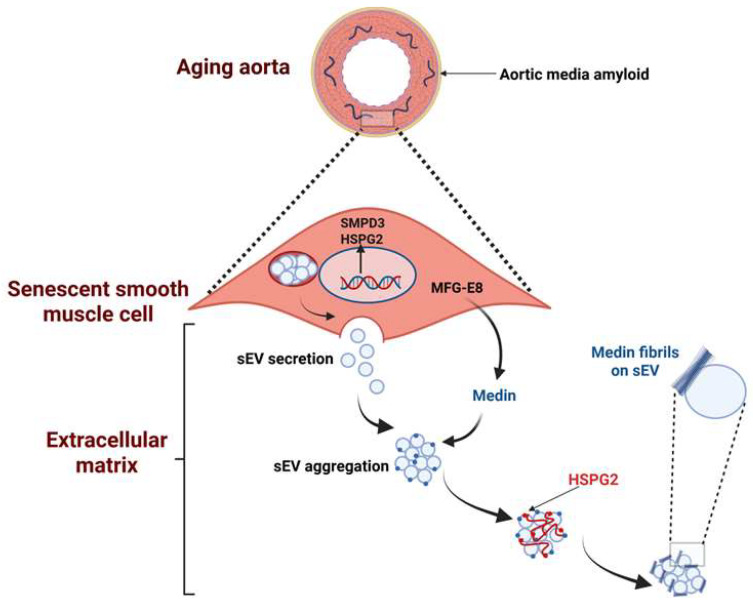
Aortic medial amyloid (AMA) is composed of medin secreted by senescent vascular smooth muscle cells (VSMCs). Small extracellular vesicles (sEVs) seem to mediate medin amyloid accumulation in the extracellular matrix (ECM). SMPD3 (sphingomyelin phosphodiesterase 3) triggers membrane curvature through sphingomyelin hydrolysis to ceramide, thereby influencing sEV release. The abundance of the proteoglycan HSPG2 (heparan sulfate proteoglycan 2) is elevated in the senescent ECM and colocalizes with sEVs and medin. Thus, VSMC-derived EVs and HSPG2 in the ECM mediate medin accumulation and AMA development associated with aging. Figure based on data from references [31,62,72]. Created in BioRender.

Medin is a soluble monomer consisting of a core created by three β-strands, with smaller and unsteady strands located at the C-termini [13], and medin aggregates are drenched with β-sheet structures, at a proportion close to 65% [12,70]. Protein synthesis in VSMCs proceeds age-dependently, and clarifying the molecular aspects of proteome variation would significantly enhance our comprehension of human biology and disease [70,71]. Senescent VSMCs enhance medin accumulation in the media via increased release of small extracellular vesicles (sEVs) resulting from upregulation of sphingomyelin phosphodiesterase 3 (SMPD3). Moreover, there is increased ECM medin on the EV surface and EV binding to heparan sulfate proteoglycan 2 (HSPG2) in the ECM [72] (Figure 2). The increased expression of HSPG2 in the senescent ECM fosters the generation of medin structures resembling medin, which differ from the small, round aggregates found in the ECM from young SMCs. Thus, senescent SMCs may generate an environment that facilitates medin aggregation and AMA formation. AMA, in turn, may affect the tissue distribution of serum amyloid A (SAA), as evidenced by experimental studies displaying that serum amyloid A fibrillation may be promoted by medin amyloid-like fibrils [73]. SAA proteins are typical elements of the human blood that are conserved during evolution, and are predominantly synthesized in the liver [74]. SAA fragments form insoluble fibrils that characterize “secondary” amyloidosis and may impair organ morphology and function, leading to organ failure.

**Figure 3:**
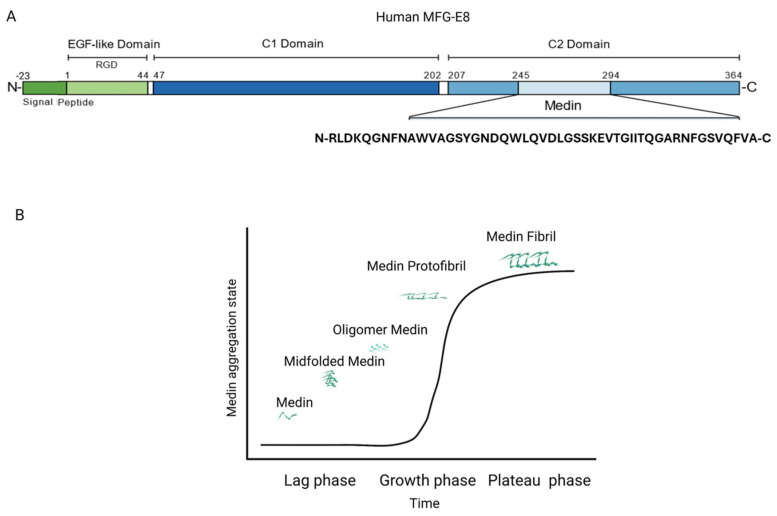
MFG-E8, medin, and medin amyloid formation. (**A**) MFG-E8, also termed lactadherin, contains an EGF-like domain on the N-terminal and two domains exhibiting homology to blood coagulation factor V/VIII (C1 and C2 domains, respectively) on the C-terminal. Medin is a peptide consisting of 50 amino acids cleaved from the C2 domain of MFG-E8. (**B**) The three-phase medin aggregation state: the lag phase, growth phase, and plateau phase. MFG-E8, milk fat globule EGF VIII; EGF, epidermal growth factor; C1, discoidin-like domain 1; C2, discoidin-like domain 2; RGD, an Arg-Gly-Asp motif. Adapted with permission from Ref. [69].

The aggregation kinetics of medin [75,76] are represented by a sigmoid curve (Figure 3) consisting of (a) a *lag phase* corresponding to monomer nucleation into oligomers and proto-fibrils, (b) a growth phase of rapid fibril elongation, and c) an equilibrium plateau generated by mature fibrils. This aggregation pattern can be found in several amyloids, such as Aβ, human islet amyloid polypeptide (hIAPP), and α-synuclein [77,78]. Although the medin monomer predominantly adopts random coil and β-sheet structures, partial helix structures have also been reported [13,79,80].

Medin and MFG-E8 interact with elastic fibers, making it likely that elastin contributes to AMA formation [81]. Computational studies suggest that the start of medin folding requires β-sheet formation around medin30–41 and medin42–50, followed by the wrapping up of other segments by their β-sheet boundaries [82]. Two medin peptides may readily aggregate into a β-sheet-rich dimer, which has a strong aggregation tendency. The dimerization of medin enhances β-sheet conformations and simultaneously leads to the generation of β-barrel oligomers. Asp (25) most likely drives the medin assembly by stabilizing the fibrillar conformation of the peptide in a way that is reminiscent of that of Asp (23) on Aβ aggregation [12].

For unknown reasons, amyloid deposits remain unrecognized by the immune system, leading to organ dysfunction. Phagocytosis assays have demonstrated that collagen associated with amyloid hinders phagocytosis and amyloid clearance [83]. In systemic light chain (LC) amyloidosis (AL), helical superstructures involving amyloid and collagen in cardiac fibrils, which likely stabilize the fibrils and decrease their clearance, have been reported [84]. Finally, in studies on mice, treatment of amyloid extracts with collagenase enhanced clearance compared with controls, coinciding with an increase in the accumulation of immune cells in the amyloid lesion [83]. Thus, collagen decreases the clearance of AMA, and dissociation of AMA from collagen might facilitate immunorecognition and clearance of pathologic amyloid deposits.

##### The Effects of Medin on the Aorta

Although amyloid fibers are crucial for amyloid pathology [85], it seems that intermediates synthesized during aggregation may be more critical for disease development. Indeed, oligomers are toxic [86] and increase membrane permeability, leading to cellular dysfunction [87]. Nevertheless, not all amyloid oligomers are cytotoxic, and those that are toxic seem to have structures that facilitate their toxicity [88].

**Figure 4:**
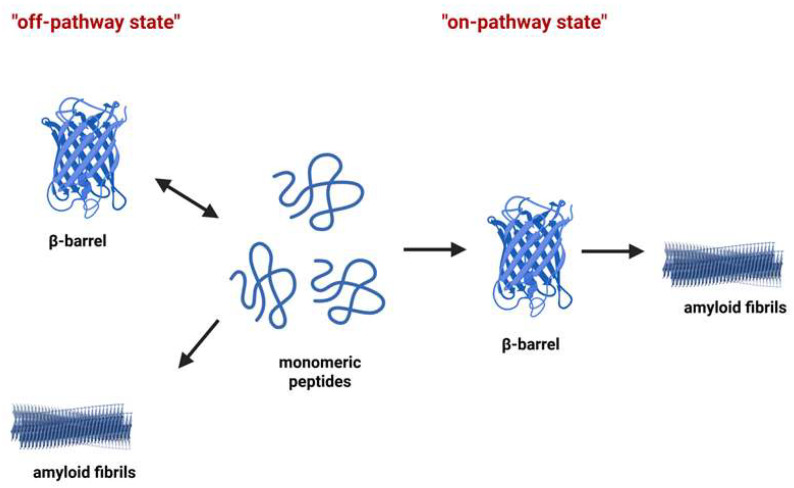
β-barrel and amyloid fibril formation. Short, differing peptides may generate stable oligomers resembling β-barrels (so-called “cylindrins”). On the pathway of fibrillogenesis, the β-barrel state can be intermediate (“on-pathway state”), or it can be formed as a result of the assembly of partially unfolded monomers (“off-pathway state”). The β-barrel oligomers are toxic and involved in the pathogenesis of various diseases. Adapted with permission from Ref. [93].

Based on the typical clinical features of amyloid diseases, it has been suggested that toxic oligomers of diverse amyloid proteins may have several morphological characteristics in common [89]. Toxic oligomers are rich in β-sheets, display an external diameter of 7–12 nm and an internal diameter of 1.5–2.5 nm, and consist of 20–60 monomers each [90,91]. Since they resemble the cytolytic toxins produced by Clostridium perfringens, which form β-barrel pores and have the capacity for membrane incorporation following the “*amyloid-pore*” hypothesis, they have been called β-barrel intermediates and considered to be the toxic oligomers in amyloid aggregation [92]. β-barrel proteins function as monomers or dimers, and additionally, they may interact with each other, forming higher-order oligomers or even aggregates. On the pathway of fibrillogenesis, the β-barrel state can be intermediate (“on-pathway state”), or it can be formed as a result of the assembly of partially unfolded monomers (“off-pathway state”) (Figure 4) [93].

Medin contributes to inflammaging by increasing oxidative stress, as evidenced by the enhancement of superoxide and reduction of nitric oxide. Autopsy studies have shown elevated MGF-E8 expression in the aortic media of the elderly, along with markers of oxidative damage. Furthermore, inflammation and intimal/medial thickening were reported in wild-type (WT) aged mice, but were not observed in age-matched MFG-E8 KO mice, which did not express MFG-E8. Conversely, experimental studies on mice demonstrated that the levels of MFG-E8 protein and mRNA were increased in the dermis during wound healing [94]. Furthermore, in the same study, MFG-E8 was observed in granulation tissue surrounding blood vessels, whereas wound healing in MFG-E8 knockout mice was slower than in wild-type mice. The findings above indicate that the physiological effects of MFG-E8 are complex and incompletely understood.

In contrast to MFG-8, medin has only toxic effects. Medin causes human EC dysfunction by reducing EC viability, migration, and proliferation, possibly by inducing oxidative and nitrative stress via advanced glycation end-products (RAGE) [95,96]. Additionally, medin may stimulate EC inflammatory signaling, leading to the production of cytokines, including IL-6 [95]. In vitro studies have also demonstrated the direct toxicity of medin to ECs [97]. Endothelial dysfunction impairs VSMC function, further aggravating the effects of aging [98]. Finally, medin-induced alterations in the ECM disrupt EC-VSMC interactions, resulting in the production of a complex mixture of components derived from ECs and VSMCs [33,99], which may influence the function of neighboring cells [100].

Interestingly, medin in its amyloid state is decreased in patients with aortic aneurysm or dissection, whereas non-fibrillar medin levels are higher in the diseased aorta [101] and correlate with increased aortic stiffness [102], suggesting that non-fibrillar forms of medin may also be toxic.

#### 2.2.3. Aortic Function

Inflammaging-induced alterations in the VSMC phenotype and in the elastin in the aortic wall cause vessel dilation, tortuosity, and stiffening that raise systolic and pulse blood pressure, enhance PWV, and lead to hyperpulsatile perfusion that damages the arterioles and promotes cognitive dysfunction (Figure 1) [103]. AMA contributes to this process by increasing inflammatory and oxidative stress, which accelerates age-related aortic dysfunction [95,97,104,105].

The aging-induced increase in AoSt occurs before the development of hypertension and may contribute to ethnic variations in cardiovascular and brain health [106,107,108]. Elevated AoSt impairs LV–arterial coupling and augments LV pulsatile load, resulting in LV hypertrophy, a significant risk factor for heart failure (HF) development [109,110]. In addition, increased AoSt impairs the optimization of the hemodynamic coupling between the LV, the aorta, and the brain [36,111]. This alters PWV dynamics, leading to increased transmission of pulsatile load, which is deleterious to the cerebral microvasculature and brain tissues. Eventually, the greater stiffness of the aorta compared to the carotid artery modifies wave reflections, facilitating the excessive transmission of pulsatile energy into the cerebrovascular network, contributing to cognitive impairments such as AD or another type of vascular dementia [112,113].

## 3. Medin, Aortic Stiffness, and Alzheimer’s Disease

Cognitive impairment in the elderly is driven by both neurodegeneration and impairment of the cerebral vasculature [114]. Neurodegenerative and cerebrovascular interactions that adversely affect cognition are seen in AD and vascular dementia (VaD) [115], with medin playing an essential role within this context.

### 3.1. Cerebral Medin

#### 3.1.1. Cerebral Medin Levels and Effects

There is evidence to suggest that cerebral medin facilitates AD development and progression. In a study that determined the medin levels in cerebral arterioles from brain donors without dementia (ND), AD, VaD, or combined AD and VaD, medin levels were higher in AD, VaD, or combined AD/VaD than in ND, with medin being the strongest predictor of AD diagnosis [116]. Further, in Aβ precursor protein (APP) transgenic mice and AD patients, medin was found in proximity to vascular Aβ deposits, and in mice, medin deficiency reduced vascular Aβ deposition by half [75]. Moreover, in both the mouse and human brain, MFG-E8 accumulated in the vessels, and expression of both MFG-E8 and medin increased in parallel with vascular Aβ. Additionally, analysis of data from the Religious Orders Study (ROS) and Rush Memory and Aging Project (MAP), referred to in combination as ROSMAP, patients with AD had higher MFGE8 vascular expression levels, which were associated with accelerated cognitive decline [75]. The same study reported that medin promoted Aβ aggregation by forming heterologous fibrils with Aβ. Thus, medin, together with vascular Aβ deposits, accelerates cognitive decline in Alzheimer’s disease [117]. Finally, another study found that both Aβ and medin are aggregation-prone, and their mixture tends to form β-sheet-rich hetero-aggregates [118]. In the same study, the formation of Aβ-medin hetero-aggregates did not limit Aβ and medin from recruiting additional Aβ and medin peptides.

Medin exerts several adverse effects on the cerebral vasculature. Medin is capable of augmenting neuroinflammation by inducing endothelial dysfunction and immune activation, as well as cytotoxicity [95]. In human arterioles, both medin and Aβ induce endothelial dysfunction and oxidative stress [95,119], suggesting the potential for interactions between these two amyloids. In human brain VSMCs, medin increases the expression of their genes and the secretion of IL-6, IL-8, and MCP-1 [120], which are potent mediators of inflammatory atherogenesis, synergistically facilitating the interaction between VSMCs and monocytes [121]. Likewise, in studies in which human brain VSMCs (HBVSMCs) were exposed to medin, pro-inflammatory activation of HBVSMCs was observed [122].

Notably, medin in the vasculature of the human brain is not stained by amyloid dyes, suggesting that amyloids can exist as distinct “strains” with differing affinity for amyloid dyes [123,124]. It seems, therefore, that there may be local amplification of different medin strains that form in the mouse and human vasculature, explaining the differences in amyloid dye affinity. In this regard, it has also been demonstrated that soluble amyloid-β monomers and oligomers significantly impair vascular function in AD [125,126,127], and it is therefore essential to identify the specific medin aggregates that contribute to vascular dysfunction in the aging brain.

Medin is also involved in the activation of microglia and astrocytes in AD, and contributes to the release of pro-inflammatory cytokines and chemokines [128]. In culture, more astrocytes and microglia phagocytose synapses were derived from AD patients than from controls, with MFG-E8 having a protective effect [129]. Similar findings were observed in VaD, in which medin in the cerebral collateral arteries and parenchymal arterioles, white matter lesion scores, and astrocyte activity were higher in VaD patients versus cognitively normal donors [130]. EC immune activation and reduced EC viability, as well as IL-8 production in the astrocytes, were enhanced after exposure to medin-treated ECs. Further, medin induces aging-associated hypercoagulability by decreasing the synthesis of the anticoagulant protein thrombomodulin by human brain microvascular endothelial cells (HBMVECs) and increasing the expression of the procoagulant proteins PAI-1 and tissue factor [131].

Usually, an amyloidosis type is related to one or two amyloid peptides or proteins [77,132]. However, in clinical practice, different amyloid peptides are often present within the same tissue or organ [133]. The formation of “seeds”, the nuclei generated by protein polymerization, promotes conversion of soluble proteins to fibrils [134]. Cross-seeding results from either seeds of the same protein (homologous) or seeds of one protein (heterologous) catalyzing the fibrillation of another protein [135]. Cross-seeding between different amyloid proteins may explain the simultaneous appearance of more than one misfolded protein in the same disease, as well as the simultaneous presence of more than one protein misfolding disorder (PMD) in the same individual [136,137]. Individuals suffering from a PMD are at high risk of developing another [138,139]. Finally, multiple amyloid diseases may be present simultaneously in the same patient (e.g., AD alongside Parkinson’s disease [140] or AMA alongside AD [116]), increasing disease severity and accelerating progression.

#### 3.1.2. Potential Mechanisms Underlying Elevated Cerebral Medin

The exact mechanism(s) underlying the elevated level of cerebral medin observed in AD are virtually unknown. However, there is some evidence implicating cerebral small vessel disease (cSVD) and the consequent hyperpulsatile flow occurring within the context of increased AoSt, which is associated both with an increase in arteriolar medin synthesis and a decrease in medin clearance via the glymphatic system.

##### cSVD and Increased Medin Synthesis

cSVD adversely affects small cells (40–200 μm) and contributes to 45% of dementias [141]. A white matter hyperintensity (WMH) burden, the predominant cSVD feature on brain magnetic resonance imaging (MRI), is tightly linked to increased AD risk [142].

The two primary forms of cSVD are arteriolosclerosis and cerebral amyloid angiopathy (CAA). Arteriolosclerosis refers to pathological processes affecting small vessels, which are susceptible to inadequately controlled hypertension or diabetes mellitus. CAA is characterized by the accumulation of Aβ and medin in the wall of small cortical and leptomeningeal arterioles and arteries [141]. Risk factors for CAA development include aging, hypertension, and carrying specific alleles of the apolipoprotein E (ApoE) gene [143]. CAA has a long preclinical phase starting decades before symptoms emerge, and it is incidentally found in approximately 16% of asymptomatic elderly individuals during MRI [144]. In ROSMAP, which included elderly men and women who underwent brain autopsy at the time of death, atherosclerosis was present in 39% and arteriolosclerosis in 35% of participants [145]. Further, the severity of both atherosclerosis and arteriolosclerosis was associated with a significantly higher risk of AD dementia [145].

In the same project, AD was the most prevalent pathology (≈65% of brains), followed by ischaemic infarcts, CAA, atherosclerosis, and arteriolosclerosis [146]. Importantly, each vascular pathology was present in >30% of brains, and when present, each was related to 20–30% of an individual’s cognitive decline associated with aging [18]. It seems that the overlap between neurodegenerative and vascular pathologies may contribute to the cognitive impairment associated with aging, as evidenced by the fact that over the last 3–4 decades, the dementia incidence in developed countries has declined, in parallel with the control of cardiovascular risk factors [147].

cSVD is characterized by increased vessel stiffness and pulsatility, white matter hypoperfusion, and reduced cerebrovascular reactivity (CVR) [148,149,150,151,152] occurring, as previously described, predominantly in elderly patients with increased AoSt.

In the Atherosclerosis Risk in Communities (ARIC)–Neurocognitive Study (n = 320, 76 (5) years, 45% black, 27% mild cognitive impairment (MCI)), the heart-carotid (hc)PWV was directly related to Aβ deposition, whereas the carotid-femoral (cf)PWV was inversely related to brain volumes in regions susceptible to AD and associated with a WMH burden [19]. A subanalysis of the ARIC Study revealed that intracranial atherosclerotic disease revealed by high-resolution vessel wall MRI was not associated with brain Aβ deposition [153].

In the Vanderbilt Memory & Aging Project, AoSt, assessed by aortic PWV measured with cardiac magnetic resonance (CMR), was related to lower regional cerebral blood flow (CBF) and higher cerebrovascular resistance in cognitively normal elderly individuals, and this relationship was stronger among those with a genetic predisposition for AD (APOE ε4 carriers with mild cognitive impairment) [154].

Finally, a recent meta-analysis of 37 studies demonstrated that increased AoSt was associated with cSVD [155]. In the same study, subgroup analysis of AoSt studies demonstrated an association with WMH [155].

From a mechanistic point of view, the increased AoSt observed with advancing age is associated with pulse pressure (PP) widening throughout the arterial tree, including in microvessels, which, in the brain, have a thin wall and lack the structure required to confront the increased PP and hyperpulsatile flow [156,157]. The latter may eventually lead to VSMC damage, resulting in increased amyloid deposition (Aβ and presumably medin) and microvascular dysfunction. As agitation is a risk factor for nucleation, we may assume that excessive pressure and flow pulsatility in the cerebral vessels could also provide a highly mobile environment, facilitating the formation of Aβ and medin amyloid [158].

##### cSVD and Decreased Medin Clearance

**Figure 5:**
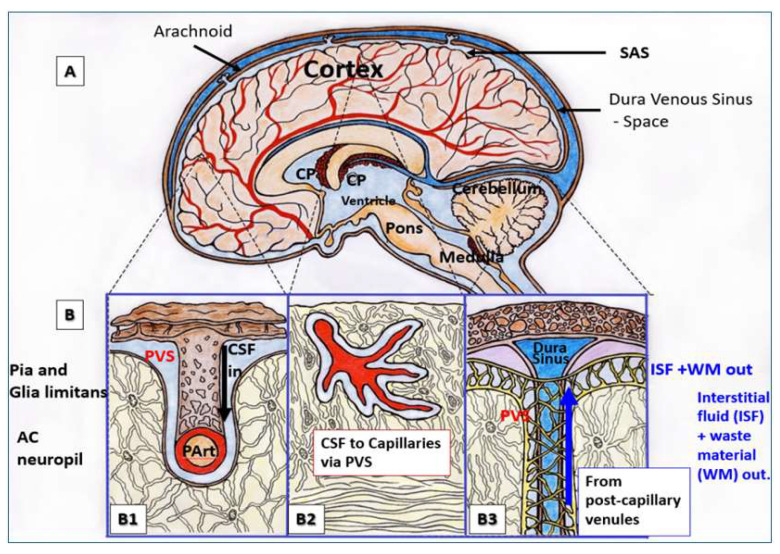
Brain perivascular spaces (PVSs). (**A**) Demarcations of specific brain regions (dashed lines). (**B**) The pia artery inside the subarachnoid space (SAS) penetrates deeper into the brain structure through adjacent PVSs, shown with blue coloration (**B1**), and horizontally–diagonally (**B2**). The PVSs allow the influx (black arrow) of cerebrospinal fluid (CSF) to the parenchymal interstitial fluid space (ISF) through the arteriolar PVS. Panel (**B3**) shows the efflux (blue arrow) of the interstitial fluid waste materials (WMs) of the pial venular PVS to the pial vein PVS that enters the subarachnoid space, which eventually drain into the dural venous sinus space. Adapted with permission from Ref. [161].

The glymphatic system is a waste clearance system in the brain that utilizes a network of fluid-filled perivascular spaces (PVSs, also known as Virchow–Robin spaces) surrounding blood vessels, formed by astroglial cells (Figure 5) [159]. Moreover, the glymphatic system distributes essential compounds (e.g., glucose, lipids, amino acids, and neurotransmitters) throughout the brain. The parenchymal accumulation of Aβ plaques and tau protein tangles, which characterizes AD, has been attributed to several factors, including an overall impairment in Aβ clearance [160].

Glymphatic function depends on arterial pulsatility, which propels the cerebrospinal fluid (CSF) through the PVS and into the interstitial fluid (ISF) [162]. Cardiovascular disorders that impair cardiac output, such as heart failure or arrhythmias, as well as conditions that increase AoSt, impair CSF flow through the PVS [163]. In elderly hypertensive patients, glymphatic dysfunction is observed, as indicated by analysis of the perivascular space index, obtained with diffusion tensor imaging (DTI) [164]. Likewise, a decrease in glymphatic transport was observed in spontaneously hypertensive rats with cSVD [165]. Significantly, enlarged PVSs are associated with poor glymphatic clearance and cognitive performance in AD [166,167,168]. From a mechanistic point of view, it is reasonable to assume that increased AoSt, by generating excessive pressure and flow pulsatility, reduces glymphatic clearance of Aβ, tau protein, and, likely, medin [161,166,169]. Admittedly, however, medin clearance via the glymphatic system has not yet been convincingly demonstrated and remains under investigation.

## 4. Clinical Implications

Monoclonal antibodies (MABs) targeting toxic Aβ aggregates, such as oligomers, protofibrils, fibrils, and plaques (aducanumab, lecanemab, donanemab, and gantenerumab), have proved effective for the management of AD patients with Aβ pathology in the early stages of the disease [170,171,172,173]. In this regard, MABs have been shown to reduce Aβ plaques and slow cognitive decline by 30% [174]. However, MAB immunotherapy is far from curative [170,175], and is associated with an acceleration in brain volume loss and ventricular enlargement [176,177], which are objective measures of disease progression and severity of neurodegeneration in AD [178]. As the mechanisms and the long-term consequences of brain atrophy induced by anti-amyloid MABs in AD remain virtually unknown, concerns have been raised regarding the possible adverse effects of this treatment modality on the cognitive function and progression of the disease. Since anti-amyloid MABs clear away formed Aβ plaques and attenuate relevant toxicity, but do not delay plaque formation by inhibiting Aβ production, combination therapies have been proposed for AD [179].

**Figure 6:**
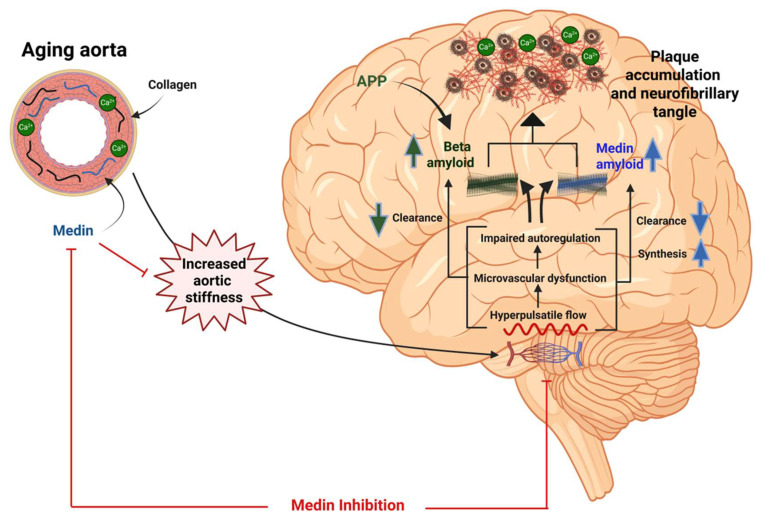
Hypothesis for the role of medin in linking aortic stiffness (AoSt) with Alzheimer’s disease (AD) in the elderly. Medin amyloid is virtually a universal finding in the aging aorta, and contributes to an increase in AoSt. Increased AoSt is associated with an increased pulsatile load transmitted in the cerebral microvasculature, which, in turn, increases cerebral medin and medin amyloid by (a) increasing synthesis via vascular smooth muscle cell damage and agitation-enhanced nucleation, and (b) possibly decreasing glymphatic clearance. Medin synergizes with vascular Aβ deposits, contributing to an accelerated cognitive decline in AD. APP (Amyloid Precursor Protein) is a transmembrane protein that plays a crucial role in brain function and is particularly concentrated in synapses of neurons, and it is implicated in amyloid plaque formation in AD. Admittedly, however, this hypothesis, although rational, requires experimental and clinical validation. Created in BioRender.

AoSt increases moderately before 50 years of age, and markedly thereafter [180]. Although AD usually affects elderly individuals aged 60 and above [181], the accumulation of Aβ plaques usually precedes clinical manifestations by 10–20 years [182]. Thus, in many individuals, an increase in AoSt may precede or coincide with Aβ plaque and medin co-aggregation and clinical AD. Theoretically, therefore, both MFG-E8 and medin inhibition may attenuate age-induced AoSt and cognitive decline, as well as reducing the incidence of AD (Figure 6). However, MFG-E8 interferes with several biological functions, including immunomodulation, cell adhesion, angiogenesis, and tissue regeneration, which may be adversely affected by its inhibition [183]. On the other hand, medin has no known physiological functions, and its inhibition seems to be an attractive and feasible treatment modality to combat aortic stiffening and prevent AD. In addition, given its co-segregation with Aβ, targeting medin may have additional benefits in the management of established AD.

## 5. Conclusions and Future Perspectives

There is converging evidence for a significant role of medin amyloid as a mediator of the age-related arterial pathology underlying both AoSt and AD. Nevertheless, crucial questions remain unanswered, such as (a) the source of medin modulating Aβ deposition leading to cognitive impairment, (b) the exact mechanism underlying clearance of medin in the brain, (c) the utility of medin as a biomarker for AD detection in the preclinical stage or the use of medin levels as a measure of AD severity, and d) whether medin aggregates increase AoSt due to physical interaction with elastin or indirectly through augmentation of inflammaging. Given the high prevalence of cardiovascular, cerebrovascular, and neurodegenerative diseases in the aging population associated with increased AoSt, the preservation of aortic function remains a major challenge in medical research. Targeting medin aggregation should therefore be investigated as a novel treatment modality to decelerate aortic stiffening and promote healthy aging.

## Data Availability

No new data were created or analyzed in this work. Data sharing is not applicable to this article.
